# Is Wilson’s religion Durkheim’s, or Hobbes’s Leviathan?

**DOI:** 10.1007/s40656-021-00375-w

**Published:** 2021-02-15

**Authors:** Andrew R. Atkinson

**Affiliations:** Society & Cognition Unit, University of Bialystok, Warminska 31/m22, 15-549 Bialystok, Poland

**Keywords:** Religion, Adaptation, Cooperation, Group-selection, Hobbes, Durkheim, Leviathan

## Abstract

This paper critically supports the modern evolutionary explanation of religion popularised by David Sloan Wilson, by comparing it with those of his predecessors, namely Emile Durkheim and Thomas Hobbes, and to some biological examples which seem analogous to religions as kinds of superorganisms in their own right. The aim of the paper is to draw out a theoretical pedigree in philosophy and sociology that is reflected down the lines of various other evolutionarily minded contributors on the subject of religion. The general theme is of evolved large-scale cooperative structures. A scholarly concern is as follows: Wilson (Darwin’s Cathedral: Evolution, Religion, And The Nature Of Society, University of Chicago Press, Chicago, 2002) draws on Durkheim, (The elementary forms of religious life. Free Press, New york, 1912) using Calvinism as an example without mentioning Hobbes (Leviathan, Edited by E. Curley, Cambridge, Hackett, 1651), but it was Thomas Hobbes (1588–1679) who used Calvinism as an example of a leviathanesque religious structure—which is not acknowledged by either Wilson or Durkheim. If there are even any similarities between these authors, there appears to be an omission somewhere which should rightly be accounted for by giving credit to Hobbes where it is due. I issue on conclusion, what it is that makes Wilson’s approach radically different to that it skates on. I also issue it with a cautionary word.

## Introduction

Religion and science, particularly to those paying attention to the science versus religion debate, have often been seen at loggerheads. An unsurprising product of the popularly perceived antagonism between the two, has been the constant need to champion the facts of evolution over creationism. Something in danger of being overlooked in that debate is the idea that religion itself has evolved, somehow, and that it might possibly be, or have been, in many ways, adaptive. That idea (c.f. Achtner 2009), of course, has indeed been pioneered by Wilson (2002, 2019) and in a similar zeitgeist, by Norenzayan (2013), and Gray and Watts (2017). This paper aims, in that same zeitgeist, to present some of the thinking behind the organismal view of religions as naturally evolved phenomena, but in the context of a pedigree of intuitions from philosophy and the social sciences. The fundamental notion underlying the naturalistic account of religion I present here, is of evolutionarily improbable large scale cooperation in *Homo sapiens*. I say ‘evolutionarily improbable’ because largescale cooperation becomes difficult to maintain beyond the confines of ‘kith’ (Queller 2011) without institutionalised structures in place to support them. Religions are thought to bind^1^ those who share concomitant cultural traits, such that those who share those traits are considered by Wilson as analogous to organisms in their own right—outcompeting other less tightly knit groups, and whatever might knit them together. Indeed, parallels to the tightly knit nature of a family appear at the larger end of the cooperative scale, such that the interests of sociologists dealing with social relationships at one position on the scale, and political scientists dealing with the structures of larger institutions at another, become formally relatable. This paper should not be read as a belated discussion of Wilson (2002), rather, as a belated acknowledgement of Thomas Hobbes’s thought in the history of ideas about the structural mechanisms that support human social arrangements. For in depth recent discussion of Wilson (2002), see Sosis et al. (2017). In what follows immediately, I (1) outline Wilsons position on religion before (2) going on argue that, for all its apparent modernity, it is in fact rooted in as far back as Plato’s *Republic*, in particular Hobbes's *Leviathan* (1651), and, of course, Emile Durkheim's, *Elementary Forms of Religious Life* (1912). In (3) I proffer some useful biological analogies for sociologists thinking about religions—ranging from slime moulds to bait balls, and social insect colonies—as displaying changing states and structures in response to certain environmental cues. In conclusion (4) I argue that though it is indeed fruitful to consider religions in much the same way as the biological entities I mention, the view that religion is ultimately adaptive deserves both credit to some previously unacknowledged thinkers, and caution before swallowing it hook, line, and sinker.

## Wilson’s religion

Arguably, the widely considered seminal contributor to the modern evolutionary treatment of religion has been David Sloan Wilson. The first of his major works, addressing religion specifically, was *Darwin’s Cathedral* (2002), and more recently *This View of Life* (2019). Much of the other modern scientific work on religion comes from cognitive scientists (Lawson and McCauley 1990; Guthrie 1993) and psychologists (Bering 2002, 2011; Barrett 2004; Norenzayan 2013) to name but a few. Wilson’s treatment of religion is strictly evolutionary^2^ and has served to further illustrate his fascination with the evolution of altruism and multi-level selectionist explanations of it (Sober and Wilson 1998). The problem of altruism with which Wilson has been concerned, is that within a world of selfish individuals, costly altruistic behaviour should not evolve. However, if one appeals to selection pressures further up the biological hierarchy then one should observe that groups of altruists will outcompete groups composed entirely of selfish individuals (for further discussion of the scope of multi-level selection, see Pievani and Parravicini 2016). Therefore, selection might act at the group level and thusly is operable at multiple levels of biological organisation—leaving open an empirical matter of which level of selection should be invoked when explaining the emergence of a given trait. The philosophical fleshing out of multi-level selection, or indeed what one means by an individual or group upon which Darwinian principles can act, has already been accomplished by a number of philosophers of biology (*e.g.* Okasha 2006; Godfrey-Smith 2009; Clarke 2010) such that, although it had previously and persistently remained controversial (Okasha 2001), multi-level selection has now been widely accepted in theory—but not so much by those schooled in the gene-centred view of evolution since Dawkins (1976). That those schooled in a gene-centric view of evolution might want to do things in a particular way, is all fine and well as multi-level selection is not taken to be an alternative, and is in fact complementary. Regardless of preference for level-of-analysis, religions can be, and perhaps should rightly be, analysed as group-level phenomena no matter which Darwinian ‘individuals’ turn out to be most relevant players. Wilson has been preoccupied with a neo-Darwinian project for much of his career, and has admirably braved the face of fierce criticism for it (see Martin 2014; Colborne 2016; Paden 2016). His most recent book (Wilson 2019) carries on in that vein and continues to address religion in terms of functional adaptiveness as a group-level phenomenon. Religions ‘manage the suite of adaptive problems related to reproduction via the costly signalling of strategic information useful for attracting, acquiring, and retaining mates, ensuring paternity certainty, preventing mate defection and infidelity, encouraging parental investment, and more.’ (Slone and Van Slyk, 2015, p 3.) Much of the criticism Wilson receives (away from *Darwin’s Cathedral*) is generally over his stance on group selection generally, irrespective of his special application of group-selectionism to religion. Following its publication, *Darwin's Cathedral* (2002) was given sympathetic attention by a number of authors (*e.g.* Diamond 2002; Tiger 2002; Benzon 2003; Falk 2003; MacDonald 2003; Sosis 2003; Mysterud 2004). Mysterud (2004) (sympathetic to Wilson's position), claimed only two reviewers had been critical or sceptical (Ruse 2002; Orr 2003). However, (Rolston 2004) followed with another critical review which concluded, ‘[T]he central thesis of *Darwin’s Cathedral* is one of those half-truths that is welcome up to a point but dangerous if (mis)taken for the whole’ (Rolston 2004, p 802). Of the two skeptical reviews Mysterud identified, Ruse’s is an exposition of Wilson's position on religion which concludes in disagreement not argued for^3^—merely because Ruse is an advocate of individual selection (a pointless objection because individual selection is embraced by multi-level selection.)

**Fig. 1:**
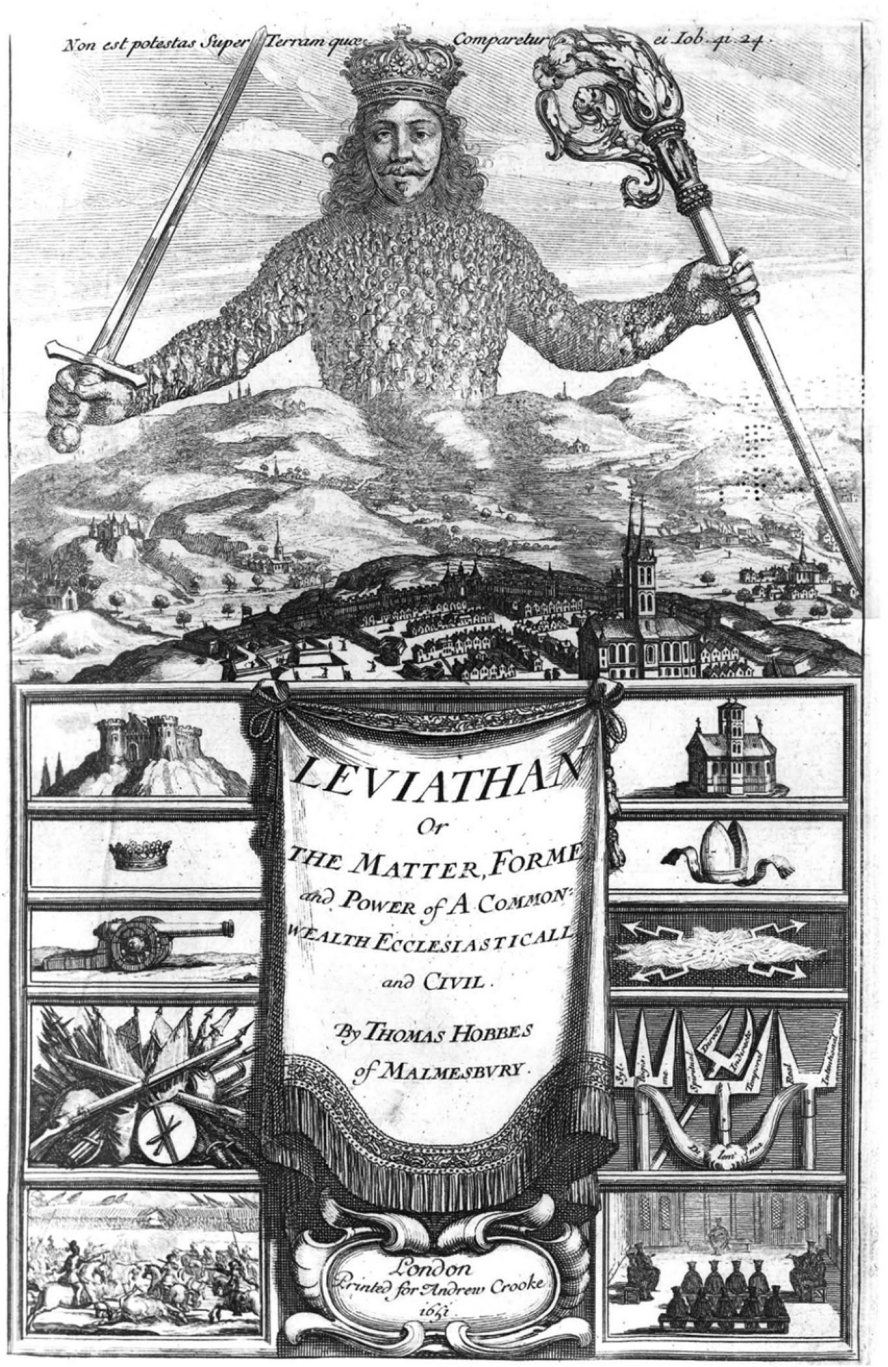
The frontispiece of Leviathan (by Abraham Bosse and designed by Hobbes himself). The body of the sovereign is made up of a collection of individual bodies

The second, from Orr, is a much more damning appraisal. Orr, a biologist, asks whether Wilson's view welcomes any nontrivial insights into religion and claims that the answer is that it does not(Orr, 2003, p 200). For Orr, Wilson's position suffers three sorts of problem. The first of these problems for Orr, is that ‘Wilson’s theory can explain just about any fact because it embraces just about every conceivable form of selection’ (Orr 2003, p 200)—indeed, for Wilson ‘it is multilevel selection theory that explains the nature of religion’ (Wilson 2002. p 119). Orr wonders whether certain features of religion are ‘nakedly selfish’ asking if those features that are good for the group are in fact bad for the individual. Orr claims that if one invokes kin or group selection, then Wilson's theory isn't strictly biological and must embrace cultural evolution.^4^ This, says Orr, is wholly uncontroversial, and nothing new. Orr also criticises Wilson for focusing on the material benefits of religion. Wilson (2002, p 162) claims that members of a religious group should prosper more than isolated individuals or members of less adaptively organised groups—but then (2002, p 168) announces that material benefits aren't the whole story. Instead, what Orr terms ‘vague psychological vibes’ are supposedly to be had of religious commitment. Orr asks if it makes sense to for poor people who might surrender a greater amount of money to churches than rich people, to say that non-wealthy tithing donors might attain that ‘feel-good factor’ as ‘hard material’ gain. Orr also complains that Wilson's proxies for fitness are frustratingly flexible stating that they are ‘sometimes biological, sometimes financial, and sometimes psychological.’ (2003, p 201) The argument from Orr is, ‘Wilson's combination of multiple evolutionary forces (individual selection, group selection, cultural evolution) and flexible fitness measures (biological, financial, psychological) seems virtually guaranteed to be consistent with the rough outlines of religious life.’ (2003, p 201) A second problem with *Darwin's Cathedral*, claims Orr, is that on examining the particulars of religious life, Wilson's theory, in attempting to explain particular features of religion, sometimes ‘slips into silliness or error’ (2003, p 201). The *silliness* Orr claims to have identified is that in spending a good deal of time on Calvinism and its adaptive traits, Wilson failed to acknowledge that Calvinism had a ‘dark side’. For example, playing games on Easter was met with prison sentences, and on one occasion a military commander was imprisoned for inappropriate dancing at a wedding,^5^ the welfare of Calvin's ‘beloved church’ all that was ultimately at stake. The issue was of course, whether or not powerful members of the community were to be held accountable to the same moral standards as everyone else. That Calvinism sought to address these issues, and as such are cited by Wilson as examples of religious adaptation, is for Orr, one of a number of insights on Wilson's part that are crushingly banal. Orr says, ‘If these are the sort of insights that follow from the multilevel selection analysis of religion, I doubt religion scholars will soon flock to their nearest evolution classroom.’ (2003, p 201) The *error* in Wilson's analysis of religion, for Orr, is thought to be found in the assumption that religion provided a mini welfare state to its members such that it provided differential rates of survival and reproduction which allowed them to outcompete the followers of other religious and non-religious doctrines.(Fig. 1)

**Fig. 2:**
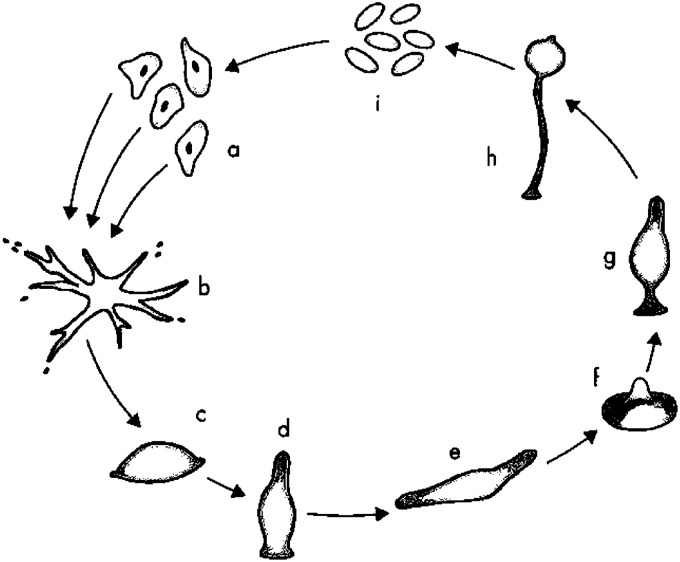
Life cycle of a cellular slime mould such as D. discoideum. **a** Free-living, individual haploid amoebae. **b** Aggregation of amoebae. **c** Mound formation. **d** Emergence and elongation of the tip section. Elongation continues until the structure falls over to form the migrating slug in **e**. **f** 'Mexican hat'. **g** Developing fruiting body. **h** Mature fruiting body with live spore cells atop the dead cells forming the stalk. **i** Dispersing spores. (Reid and Latty 2016) (Drawing courtesy of E.J.T. Middleton)

A third and final problem Orr wishes to raise he terms a ‘certain arbitrariness’ which ‘seems to characterise what Wilson deems suitable subject matter for multilevel selection theory. Why can't multilevel selection explain science too? A simple answer to Orr here might be that it does. Orr claims scientific group endeavour can fulfil all the requirements desired by such explanation. Scientists accomplish more as a group than as individuals—along with those who enjoy its technological innovations, it rewards its practitioners with material riches, it demands adherence to a set of beliefs, and (Orr’s example is that it's wrong to make up data) it punishes cheaters and freeloaders, even blessing its practitioners with a sense of belonging. Orr rightly points out that science is the product of series of intellectual endeavours—‘not a mildly embarrassing epiphenomenon that evolved because it brings you and me material rewards’. But is it right to weigh off religious-group selection against the fecundity of science? Perhaps—perhaps not. Perhaps too, one can view the distinct branches of the special sciences as phylogenies of the offspring of natural philosophy, in much the same way religious groups bifurcate and branch off into variant daughter religions.(Fig. 2)

If Wilson is right, the possibility of cultural group selection, together with the premise that cultural traits have a bearing on genetic fitness, means ‘group selection’, *is* a significant force insofar as it acts on culturally distinct groups. (Richerson and Boyd 2005) concurred. They said;

Group selection on cultural variation has been an important force in human evolution. Conformist bias and rapid cultural adaptation conspire to generate oodles of behavioural variation between groups. The conformist effect overcomes the critical problem with group selection. (p 163).

A multi-level selectionist ethos still lives on (e.g. Turner et al. 2018; Kavanagh 2019 for a review). Now I want to briefly address, for sheer retort, what is going to be an obvious objection from the religious polemicist—Why would evolution select for any trait which supports belief in that which is palpably untrue? (On multi-level selection and evolutionary epistemology see De Cruz et al. 2011; Bradie and Harms 2020)A striking feature of Wilson’s view on religion is that he sees within his evolutionary development of individual cultures, an idea he captures by the terms *factual* and *practical* realism. It is an assertion any ardent atheist is going to want to ask about. Surely scientific epistemology should supplant fallacious religious efforts mixed into an enterprise of the factual determination of ‘the truth’. Wilson's answer to this question is unsettling but, I argue, very well founded from an evolutionary perspective.

Perhaps what seems to be an adversarial relationship between believers and nonbelievers in fact represents a healthy balance between factual and practical realism that keeps social groups as a whole on an even keel. (2002, p 229).

By this Wilson seems to be admitting *two* views that have played alongside each other for opportunity in shaping our evolutionary development as a species—*truth*, and *falsity*. It is assumed here, that by ‘factual realism’, Wilson means *truth*, and by ‘practical realism’, he means—‘*nothing* of the sort’, *and*, that these can come into competition with one another insofar as one may be more adaptive than the other in a given context. Where the factual realists might object to the unreality of religious foundation, Wilson has to say;-

Religious belief is not detached from reality…. [R]ather, it is intimately connected to reality by motivating behaviours that are adaptive in the real world—an awesome achievement when we appreciate the complexity that is required to become connected in this practical sense. (2002, p 228).

It is unlikely therefore, that an *untrue* idea, would persist down evolutionary lines if it is in any way dysfunctional—so, *function* can be ascribed even to false religious beliefs. Wilson goes on to say then, that;

If there is a trade-off between the two forms of realism, such that our beliefs can become more adaptive only by becoming factually less true, then factual realism will be the loser every time. To paraphrase evolutionary psychologists, factual realists detached from practical reality were not among our ancestors. It is the person who elevates factual truth above practical truth who must be accused of mental weakness from an evolutionary perspective. (2002, p 228).

A criticism I wish to raise is that Wilson’s account of religion doesn’t explain *how* religions have God[s]. Wilson can’t derive God by evolutionary means—and only ascribes function to acquiescing in the belief. However, “God” is indeed a vital concept in effecting the sort of prosocial behaviour Wilson claims is selectable for. (Okasha 2003) raises similar objections;

if the adaptive value of religion consists in the prosocial behaviours that religious believers display towards each other, why do virtually all religions require their members to adopt bizarre beliefs about supernatural deities? Can such fantastical beliefs really be adaptive for the community of religious believers? (p 702).

Wilson’s theory contributes in some way to pinning the origin of religion as resting on the evolutionary advantages of cooperative behaviour. However he has *not* had much to say about the origin of the idea of God other than to say that once we’ve got it, it might be adaptive to believe in. A functionalist account of belief in “Big Gods” came from Norenzayan (2013) and again from Gray and Watts (2017) where the effects of such belief are described to augment the kind of cooperative behaviour Wilson purports to bind his religious organism together such that, it seems*, “Si Dieu n'existait pas, il faudrait l'inventer”,* ‘…if God did not exist, it would be necessary to invent him’ (Voltaire 1770). Explanations of the origins of God concepts come from the cognitive science of religion centering on the role of hyperactive (or hypersensitive) agency detection and theory of mind in the generation of important key religious concepts (see Atkinson 2020 for a discussion). But the question from Okasha (2003), and the functional role of a God-like sovereign being in all this, *is* one which does seem to have been of interest to Thomas Hobbes, some 350 years before Wilson got going with his treatment of religion. I would like to turn, at this point, to some interesting parallels to Wilson’s religion, in arguing that there is a philosophical and sociological pedigree in the modern evolutionary synthesis of a neo-Darwinian approach to religion.

## A functionalist pedigree

Philosopher and psychologist William James, in 1880, was arguably enamoured with a Darwinian perspective on social phenomena in a similar vein to Wilson in more recent times.^6^ James said:

A remarkable parallel, which to my mind has never been noticed, obtains between the facts of social evolution and the mental growth of the race, on the one hand, and of zoological evolution, as expounded by Mr Darwin, on the other. (James 1880, p 441).

Therefore, regardless of its sophisticated evolutionary logic and contemporary neo-Darwinian language, there have been similar cooperative-social theories from a number of thinkers prior to Sober and Wilson (1998); and Wilson (2002).

The idea that human groups resemble organisms, can be found in ancient India where Hinduism’s four major castes were described as having descended from the different body parts of a God-created giant. The term ‘superorganism’ was first used by Herbert Spencer in the nineteenth century to describe human groups (Kesebir 2012, p 234). Kesebir (2012) notes that likening human societies to beehives runs from Aristotle to Shakespeare. Plato (earlier than 347 BC) also imagined a similar utopian cooperation in his *Republic,* but Plato did nothing like adding a sovereign being into the picture to help make it work. Similar thought is even found in Hegel (1832 p 457:345). Hegel says;

The saying that human beings are by their very nature free is a principle of infinite worth. But if we stick with this abstraction alone, no organic political constitution can emerge, for that requires an articulation in which duties and rights are delimited. That abstraction leaves no scope for the inequality that has to come in if a [social] organism, and with it genuine [social] vitality, is to come about.^7^

That Wilson’s treatment of religion has a select (in some places unacknowledged) pedigree in the history of human thought is only a minor complaint. Moreover, it is not just socio-evolutionary thinking about religion for which a philosophical pedigree can be found. In the cognitive science of religion, or for theories of cultural transmission, precursory thinking can also be found hundreds of years before. Arguably predicting the ‘cognitive-naturalness-of-religion-hypothesis’ (McCauley 2011), and the notion of ‘minimally counterintuitive narratives’ (Norenzayan et al. 2006), and the idea of ‘rogue cultural variants’ (Boyd and Richerson 1985) or virulent ‘memes’ (Dennett 2007), philosopher John Locke in his *Essay Concerning Human Understanding* (1690) said;

Men… can scarce avoid having some kind of ideas of those things, whose names, those they converse with, have occasion frequently to mention to them: and if it carry with it the notion of excellency, greatness, or something extraordinary; if apprehension and concernment accompany it; if the fear of absolute and irresistible power set it on upon the mind, the idea is likely to sink the deeper, and spread the farther; especially if it be such an idea, as is agreeable to the common light of reason, and naturally deducible from every part of our knowledge, as that of God is. (Locke 1690, p 71).

Wilson (2002) does acknowledge Durkheim throughout much of his thought, quoting him the first of three times at ‘religion is a unified system of beliefs and practices relative to sacred things... which unite into one single moral community called a Church, all those who adhere to them’ (p 47). What Wilson did throughout *Darwin’s Cathedral* was to pluck out what was advantageous to collect in what Durkheim said exactly in 1912, and subject it to a modern evolutionary description. It is reasonable to assume, therefore, that Wilson’s view of religion is Durkheim’s view ‘souped-up’.

Durkheim’s major insights were of course informed by pioneering comparative religion scholar William Robertson Smith’s ground-breaking works (1846–1894). Durkheim himself declared his debt to Smith’s work (see Maryanski 2014). However, I urge the reader to consider how dramatically similar the structure of Hobbes’ leviathan is to the structure of the organised religions Wilson claims to have picked out in a fresh light—and then wonder why no such link is ever made explicit. Where there is no doubt that Wilson was influenced by Durkheim, there is also no doubt that Durkheim must have been influenced by Hobbes (Follert 2020). Hobbes, in *Leviathan* (1651) argues that civil peace and social unity are best achieved by the establishment of a commonwealth through social contract. The commonwealth Hobbes describes is ruled by a sovereign power responsible for protecting the security of the commonwealth and granted *absolute* authority to ensure a ‘common defence’. Hobbes describes this commonwealth as an ‘artificial person’ (p 3) and as a body politic that mimics the human body (Copp 1980). The frontispiece to the first edition of *Leviathan,* which Hobbes helped design, portrays the commonwealth as a gigantic human form built out of the bodies of its citizens, the sovereign as its head. Hobbes even explicitly outlines the compatibility of Christian doctrine with the socio-political system of the *Leviathan* (see Martinich 1992). Moreover, for Hobbes, the Christian faith was so neatly bound up with how the sovereign state purportedly functioned, that where it was seen to be antithetical was an indication of false, primitive, inherited pagan beliefs.

A scholarly concern should follow from the fact that Wilson draws on Durkheim using Calvinism as an example without mentioning Hobbes—but it was Hobbes who drew on Calvinism as an example of a leviathanesque religious structure. This is not acknowledged by either Wilson or Durkheim and there is no reason to suppose that both authors were unable to trace the history of ideas that far back. It is absolutely certain that Durkheim was intimately aware of Hobbes’s body of work in light of the rediscovery of Durkheim’s (2011 [1894–1895]) lectures on Hobbes’ *De Cive* (Follert 2020). If there are indeed similarities between these authors, there appears to be an omission somewhere which should rightly be accounted for by giving credit to Hobbes where it is due. There are indeed direct parallels to Hobbes there in Wilson (2019). Wilson says;

We seldom associate politics and economics with religion and spirituality, and in many ways we feel the need to keep them apart, as with the separation of church and state. Nevertheless, words such as “corporation” (derived from the Latin for “body”) and phrases such as “body politic” signify that whatever we mean by the word “organism” can be applied to entities that are larger than organisms, such as a human society or a biological ecosystem. (Wilson 2019, *Prologue*).

Therefore, credit is due to Hobbes, and what can only be considered a perpetual and persistent intuition of the wider social function of peculiar institutions such as religion, and likely, the very reason religion and state politics are constantly intertwined despite best intentioned attempts to separate them. Unravelling that evolutionary tapestry^8^ is not likely to be done by quick and inelegant argument, and will perhaps require a great deal of patience on the part of the militant atheist.

The functional role of belief in the sort of supernatural deity both Hobbes and Durkheim would have been concerned with, something Wilson doesn’t arrive at by causal means (even by 2019), has been described by Norenzayan (2013), and Gray and Watts (2017). Again, and just as surprisingly, Wilson does not mention Norenzayan, or Gray and Watts. Regardless, the general argument Norenzayan makes in (Norenzayan 2013) is that under the watchful gaze of a supposedly omnipresent, omniscient, and morally concerned ‘Big God’ deity, the very sorts of supernaturally policed cooperative behaviour by which Wilson purports religion to function as adaptive, are augmented such that big God religions outcompete the millions of others nobody believes in anymore and gone extinct. That idea of God is both an incidental and cognitive by-product in origin that has evolved into the sophisticated watchman in the sky with which most are familiar (Boyer 2001; Barrett 2004; Bulbulia et al. 2013). At any rate, it is not Wilson’s concern to account for ‘how or why’ belief in Gods, but merely to describe the adaptiveness of peripheral arrangements surrounding such beliefs.

Having sufficiently established a pedigree to Wilson’s thinking on religion, in what follows immediately, I proffer examples from nature that might be thought of as sorts of group arrangements to which religious groups might share analogy. I do so in support of Wilson’s consideration of religion. I stop to observe however, that it is important to consider the ‘changing states’ of these biological examples because religious groups change in state too—religions are after all, a body of individuals with fluctuating interests. Every once in a while, a Leviathan goes to war, limbs move and grow, and perhaps even changes its head—Leviathans are peculiar beasts indeed, but achieving the peculiar is not as feat beyond the reach of evolution by natural selection at all.

## The United States of religion

Now I want to turn to analogies of religion to kinds of organism or superorganism. I do so in support of Wilson’s consideration of religion at the group level. I go on to argue that it is important to consider the changing states of these examples because religious groups change in state too—religions are after all, a body of individuals with fluctuating collective interests—and those interests may put them at odds such that they may have to form a stampede from time to time, be that to attend ritual functions or unite in battle.

Wilson says, ‘religious believers often compare their communities to a single organism or even to a social insect colony’ (2002,p 1). That Wilson compares them so is arguably useful to do, even though a great many religious believers might reject the analogy.^9^ Mormonism may indeed make use of the beehive symbol, but that Zen Buddhist monasteries were constructed to resemble a single human body seems to be clutching at straws in claiming that that's how they really thought of themselves. Despite possible complaint from religious believers themselves, religious groups *are* a body of individual organisms, and their respective religions are a niche within which they seem to flourish. Not only is the social superorganism view of religions worth consideration, so are biological examples to which they might be analogous and those don’t necessarily all occupy positions high up the biological hierarchy. For example, Dennett (2007) is fond of illustrating his selfish replicator analogy of religious information (such that his *meme* ‘hijacks’ the brain of the religious host) by talking about the ‘lancet fluke’ *Dicrocoelium dendriticum*. The lancet fluke is a parasite flatworm that hijacks the brains of ants, causing the host ant to repeatedly climb up to and fall off the tips of blades of grass where it (at the tip of the blade of grass) is more likely to get into the stomach of a sheep or a cow by ingestion—thusly inducing extremely costly suicidal behaviour. The analogy serves to illustrate the selfish replicator or virus analogy well, but it really is not near enough like the *leviathanesque* state[s] of a religious group with which we are here concerned. The Mormon use of the beehive symbol in Utah, the ‘beehive state’, has already been mentioned—whomsoever the Mormon queen might be is another matter. To boot, *Homo sapiens* systems of cooperation are flexible whereas the arrangements in a beehive are not. Whereas overnight, *Homo sapiens* can overthrow the queen and establish any kind of political dictatorship or even a democratic republic, bees cannot.

Other examples do a much better job of illustrating religious states of affairs. For example, coral groups are a *body* of thousands of *individual* polyps. The spatial competition endured by coral is the result of the competition for vital sunlight. Inevitably, the corals begin to overgrow each other potentially blocking the other’s light. They do not just prevail in the same way trees might do by overgrowing one another—coral actually aggressively *eliminate* the potential competition. When neighbouring corals get too close, they detect one another’s presence chemically. The polyps extrude their guts and digest their rivals alive. The only evidence of the loser having existed is the white limestone skeleton left behind over a ‘border dispute’, perhaps leaving territory wide open to colonisation by other forms of life (Rasher and Hay 2014). The winning coral is ‘built’ on the foundational forms of that set in place to receive the light— something like religious warfare where one or more religious groups come into conflict (Bruneau et al. 2012), and is particularly well illustrated by the history of structures like the Mosque-Cathedral of Cordoba, in Spain. The winning coral grows upon the deadened structural form of the other group, and new coral grows on the previous structural success of its rival’s pursuit of the light. But what about responses to threat? Members of a religious group may have cause to rally round the flag, crusade, and form defensive patterns of defence or attack. Such states can be found in fish. Fish have seemingly adopted behaviour to avoid being eaten by literally forming a masse swirl or ‘bait ball’ to disorient predators. Schooling fish are particularly in danger of being eaten if they are separated from the school—so they form nucleated ‘bait balls’. A bait ball serves as a method of confusing a predator (Milinski and Heller 1978). It becomes difficult for predators to pick out individual prey from fish groups because the many moving targets create a sensory overload of the predator's visual system. Milinski and Heller's findings have been demonstrated by experiment (Jeschke and Tollrian 2007) and computer simulation (Krakauer 1995). ‘Shoaling fish are the same size and silvery, so it is difficult for a visually oriented predator to pick an individual out quick enough from a mass of twisting, flashing fish, in order to grab it before it simply disappears into the shoal’ (Moyle and Cech 2004, p 195). Essentially, the tactic is neatly captured by the terms ‘confuse and duck’. By ‘duck’ I make obeisance to the idea that there is an interesting degree of arguably selfish behaviour in the bait ball such that what might appear as a united group ‘standoff’ could just be the illusion, or fortunate by-product of individual self-preservation—the risk of being eaten is greater on the periphery and decreases toward the centre. Low-risk positions at the centre of the bait ball (where there is a fight to obtain such position) will be occupied by the stronger fish (or the most selfish), whereas, subordinate animals will be forced into higher risk positions. Genic selection takes place even at the level of the bait ball. I argue that bait balls have parallels to religious groups. For most of their lives, religious group members are scattered. Though the religious synchronise regularly during ritual behaviour, they also ‘rally round the flag’ in the face of adversity. Religious groups change their state just like shoaling fish. In the face of war, for example, it is doubtful that the ancestral members of the successful religious groups of the present simply scattered and hid. I believe this ‘changing of state’ is important when considering religions from a biological perspective.

In another scenario, ants can be seen to ‘drown themselves’**,** their bodies forming a bridge for the rest of the colony to traverse—but is this interpretably altruistic act the mere happenstance of each individual trying to get across the water just resulting in a bridge of failed attempts over which to traverse the impossibly non-navigable, or is such interpretable altruistic self-sacrifice genetically encoded for the good of the group? One has to ask if this is strictly an adaptation, or just incidental fortune for those ants that avoid drowning by walking over the corpses of their dead comrades. If so, it would appear nice guys do not win out. Analogy to the ant colony is common in the literature on social evolution—but it must be pointed out, that there aren’t ‘strictly’ sterile workers in religious groups—on the contrary Blume et al. (2006) and Blume (2009) repeatedly found that religious affiliation generally increases fertility. A few case examples of celibacy are there in some religions, but nothing like as many as those in the service of the ant queen. The point about worker sterility is thought to relate to altruistic behaviour in religious groups where one or more members are in the service of others—or—as lending itself to explanations of the evolution of altruistic behaviour generally. Worker ants, in service of the ‘queen’ might put one in mind of the celibate hierarchy of the Catholic church—but that hierarchy puts such celibacy in reverse order, such that priestly or papal celibacy facilitates, somehow, the subservience of the workers.

As mentioned, an interesting observation to be had of some of the biological examples proffered here is their changing states. A large percentage of the time, the various members of these groups operate with seemingly individual selfish interests, under certain circumstances, united by a collective set of interests. It is for this reason I would like to talk about an ‘individual’ who’s changing state is something quite remarkable indeed. If *this* thing can evolve—*and* be thought of as an organism in its own right—then it is quite possible indeed to consider religious groups as either analogous to it, or entirely plausible that they are biological organisms in their own right.

Consider the changing states of the slime mould illustrated in the diagram above. That entity, is largely a considerable number of scattered amoeba/spores living and feeding individually. When food sources become scarce however, they manage to aggregate (always finding their way back to each other) to form a large mobile ‘slug’ which can move off on its own to find a new food source—whereupon, the spores are re-dispersed from a sporangiophore structure to lead individually scattered lives again. This is something akin to responding to environmental problems, then an exodus, a search for new lands, establishing a church, and going about daily life with your brothers and sisters—rinse and repeat, in ritual fashion. The bait ball too is only a state which arises under threat of predation. The coral is always expanding, and only comes to blows with other coral if they compete over the same space—with religions, all it can take for all-out war is battle over some small symbolic space—such as that by the Dome of Rock mosque in Jerusalem. Religious group’s members thusly change states in familiar ways (from a biological perspective). Thusly, cases of religious ritual and behaviour (another functional state of this religious leviathan) lend themselves to biological analogy. Perhaps it isn’t always clear what much of religious behaviour is supposed to achieve, but even fancy religious ritual states do not escape scientific explanation. Such costly signalling has been explored by (Henrich 2009; Xygalatas et al. 2013 and many others).

So it seems, if anything, that philosophical fodder is there in analogies of religious states of affairs to the naturally bizarre. Indeed, we should not forget, that human states of affairs *are* bizarre. Any alien scientist studying earth’s ecology would likely be very puzzled indeed and ask of our species; ‘Why […] do many [of their] cultures devote huge fractions of their limited resources to placating […] Gods, given that there are no Gods to placate?’ (Sterelny 2006, p 147).

## Conclusion

Although Wilson’s treatment of religion is in *some* ways nothing new, and though his lack of critical engagement with academic, non-theological, and historiographically-based religious studies is a problem for some (*e.g.* Colborne 2016), it has nevertheless been the most pioneering of its ilk. What makes his kind of approach more sophisticated or radically different than those of its predecessors is that it is informed by modern evolutionary expertise.

Modern evolutionary expertise frames the functional role of various religions within the broader context of group dynamics, without needing to stop and explain why Mohammad believes *a* or *b* or, why Ganesh is blue. This is done without needing (or wanting) to endorse the truth or falsity of any one religious view over another, or act as a proponent of any one side of the science versus religion debate. Fundamentally more important, is that Wilson’s treatment of religion is probably, along with the cognitive science of religion, one of the most relevant in the evolutionary arena, for its treatment of religion describes not just an evolutionary by-product (Boyer 2001; Atran 2002; Bering 2011) but a much richer complex of functional adaptation replete with fascinating cultural phylogenies. However, it should indeed be mentioned, it seems, that Wilson does not give credit where it’s due to Thomas Hobbes—and it is indeed peculiar that he should adopt similar examples to Hobbes’s in the *Leviathan* (1651) to discuss Durkheim while neither author really acknowledges that pedigree. It is likely an oversight, and more likely that the parallels are obvious enough such that perhaps it wasn’t deemed necessary to mention. It seems fairly logical to assume, that if Wilson is right, and Durkheim, and Hobbes, then they are each talking about the same, or very similar leviathan. If not, then they at least share some common ground indeed.

Treating religions as having a function irrespective of their truth value (Talmont-Kaminski 2013) indicates some ingenuity on Wilson’s part and I argue he is correct in adopting said stance. His assertions may still only mean that false religious belief in God[s] may have been adaptive in the past but not *necessarily* in the present. For example, ancient religious beliefs may come into conflict with modern science to the detriment of the species on a number of levels. A further point to make draws analogy to the overbearing antlers of some deer. If religion promotes evolutionarily improbable large scale cooperation such that the species becomes overly successful, then those groups may get so large that there aren’t enough resources maintain themselves, along with secularised groups with more moderate birth rates. To see religion as *ultimately* ‘adaptive’ may be unwise, therefore, if based on the assumption that religion’s function is to ever promote larger scale cooperative breeding colonies of like-minded individuals. As mentioned, the idea of multi-level selection is not without its critics, and has only been theoretically approved of (see Okasha 2006 for a mathematical application to the theory). If it has only that kind of approval, what remains as fact is merely that religion evolved—after all evolution is merely change over time, and religions do just that.

The mere idea that religion evolved, and may or may not have been adaptive, might come as a shock to those on either side of the science and religion debate, and that much in Wilson’s approach is indeed characteristically antagonistic, but, could indeed provide a common ground on which to discuss those things in religion which are adaptive, and those which are deleterious. For example, the dogmatic aspects of religion, although intended to preserve some tradition, may be deleterious, whereas some may find a way of defending religious societies as embracing innovative cultural change—*i.e.* they are able and willing to adapt in some areas, which, if anything, history has shown us and is precisely the sort of thing Wilson (2002) has argued. A capacity for cultural change is not particular to religion however, but to culture in general, and while the point is made that religions can and do make use of cultural innovation, there are areas where religions in particular dogmatically resist change. Where religions are tightly bound with a state, change may come less easily, because they are not in competition. However, where disestablishmentarianism is the general policy, religion may be forced to compete with secularising social values, and adapt accordingly or potentially perish. The religions which have survived the test of time, however much they might have resisted change, have managed to evolve. Wilson’s way of showing it is just one level of analysis amongst many which study religious individuals. Moreover, a multi-level approach to understanding religion is not exclusive of any of the other kinds of approach. Wilson’s approach therefore increases our understanding of this quite natural phenomenon, by addressing one aspect of its evolutionary dynamics. The more times the nail has been hit on the head the better. That is not to say, however, that everything is ‘hunky dory’—concerns about the effects of religion and/or why it is ultimately here are genuine and warranted. Just because something evolved, doesn’t necessarily mean it’s a good thing (Ambasciano 2019, xv). Though there may be a number of biological analogies to religions which resemble its complex and varying organisation, there may be more than the few mentioned here of even greater interest to the social sciences. For example, the sort of selfish memetic replicator or virus (Dennett 2007) describes religion as, may have chameleon-like qualities in that it can camouflage itself against the background of many areas of life such as politics, law, and economics—perhaps too for its own survival. In 2010, Susan Blackmore reneged on her view that religion is a virus, on being presented with ‘data’ from Michael Blume showing that religious individuals, regardless of education or social status, enjoy higher birth rates than their secular counterparts (Blume et al. 2010; Blume 2009; Blackmore 2010). Where ‘number of offspring produced’ is taken as a measure of fitness, that would seem to suggest that religiosity is adaptive. However, Blackmore did not consider, that one of the effects of her religious virus, might have been to effect irresponsible breeding patterns associated with overpopulation and concomitant climate change.

## Footnotes

1 Wilson claims the term religion comes from the Latin *religio,* indeed meaning ‘to unite or bind together’ (Wilson 2002, p 220). However, there are alternative etymologies and interpretations, as Wilson himself acknowledges (*ibidem*). For instance, while Wilson's preferred reading follows the famous social explanation offered by Cicero (*relegere*, from *De Nat. Deorum* II xxviii 72), we also have other Latin etymologies which implied different nuances and meanings ( e.g. *relinquere*, *religare*; see Hoyt 1912; Borgeaud 2013).

2 Whereas an evolutionary psychologist or cognitive scientist might study how the mind evolved religiosity, this is different from an examination of how religions themselves evolved and changed over time.

3 I acknowledge general argument elsewhere on the subject of group selection—a full exposition of which would go beyond the remit of this paper.

4 I side with Orr on that criticism of Wilson (2002), as the groups selected for are indeed culture groups and *must* therefore incorporate theories of cultural transmission and inheritance: memes, cultural variants etc*.* There is only one fleeting mention of memes in Wilson (2002, p 53), but to his credit, by Wilson (2019), he fully embraces cultural evolution *and* its mechanisms as a Darwinian process.

5 Orr notes that the nature of the dance is not known.

6 Indeed, Wilson’s own position may well have been influenced by the current of American pragmatist philosophy, however, this parallel comes with a caveat: James "oscillated between thinking that a 'study in human nature' […] could contribute to a 'Science of Religion'and the belief that religious experience involves an altogether supernatural domain, somehow inaccessible to science but accessible to the individual human subject" (Goodman 2017). In other words, a case could be made for James to be considered as a non-Darwinian accommodationist who entertained pro-paranormal and supernatural views (see Ambasciano 2019, p. 32 and 181, note n. 10).

7 Hodgson, the Editor of the volume, states in the footnote to this passage from Hegel, that ‘Hegel here seems to construe a social organism on the model of a natural organism, whose vitality results from the interplay of unequal parts with distinct functions.’ (p 457).

8 The intertwined relationship between religion and society was indeed Durkheim’s own position.

9 The religious themselves may each just feel to be individually theistically correct on the whole.
